# Temperature Dynamics of Porcine and Human Lungs During Static Ice Storage: Ice Is Not 4 °C †

**DOI:** 10.3390/jcm14062127

**Published:** 2025-03-20

**Authors:** Ismail Cenik, Jan Van Slambrouck, Annalisa Barbarossa, Xin Jin, An-Lies Provoost, Pratik Patel, Lucas Churchill, Ben Bulka, John Haney, Laurens J. Ceulemans

**Affiliations:** 1 Department of Thoracic Surgery, University Hospitals Leuven, 3000 Leuven, Belgium; ismail.cenik@student.kuleuven.be (I.C.); jan.vanslambrouck@kuleuven.be (J.V.S.); annalisa.barbarossa@uzleuven.be (A.B.); xin.jin@kuleuven.be (X.J.); an-lies.provoost@uzleuven.be (A.-L.P.); 2 Laboratory of Respiratory Diseases and Thoracic Surgery (BREATHE), Department of Chronic Diseases and Metabolism, KU Leuven, 3000 Leuven, Belgium; 3 Paragonix Technologies Inc., Waltham, MA 02451, USA; ppatel@paragonixtechnologies.com (P.P.); lchurchill@paragonixtechnologies.com (L.C.); bbulka@paragonixtechnologies.com (B.B.); 4 Department of Cardiothoracic Surgery, Mayo Clinic, Jacksonville, FL 32224, USA; haney.john@mayo.edu

**Keywords:** cold ischemic storage, lung transplantation, static ice storage

## Abstract

**Background**: Static ice storage (SIS) has long been accepted as the conventional lung preservation method, assuming to maintain 4 °C temperature. Although never directly confirmed by temperature measurements, this assumption has been widely accepted. We aimed to measure lung and preservation temperature with SIS in porcine experiments and clinical observations. **Methods**: Porcine lungs were preserved with SIS for 8 h (group I, *n* = 3) and 3 h followed by 10 °C storage (group II, *n* = 3). Tissue (tissueT°), first bag preservation solution (psT°) and second bag saline (salineT°) temperature were monitored. In clinical lungs (*n* = 4), psT° was monitored during SIS. Surface (surfaceT°) and core (coreT°) temperature were assessed before and after SIS (*n* = 62). **Results**: After 4 h in porcine lung group I, average tissueT° was 0.66 °C, psT° was 0.12 °C and salineT° was −0.02 °C. After 3 h in group II, average tissueT° was 1.90 °C, psT° was 0.57 °C and salineT° was 2.33 °C. In clinical observations, psT° was below 4 °C after 9–13 min and 0 °C after 78–267 min. After SIS, median surfaceT° was 1.25 °C (min-max; −3.2–9.2 °C) and coreT° was 1.45 °C (−0.4–4.8 °C). **Conclusions**: SIS leads to rapid temperature drops below 4 °C, approaching 0 °C within 2–4 h. The post-SIS lung temperature shows considerable variability and does not consistently remain at the commonly assumed 4 °C, posing potential freezing injury to donor lungs.

## 1. Introduction

Lung transplantation (LTx) is a life-saving procedure for patients with end-stage lung disease, offering extended survival and improved quality of life [1,2]. One of the key determinants of successful LTx outcomes is the cold ischemia time (CIT), the period during which the donor lung is cold-preserved at low temperature with no active perfusion. Static ice storage (SIS) has been the primary method of managing the cold preservation, involving storage in an ice-filled box [3,4,5]. Despite its global and long-standing use, current practices with SIS are not standardized and have several drawbacks. A common assumption is that SIS maintains organs at 4 °C, based on studies from over 30 years ago [6,7,8]. However, recent advancements in precision temperature measurement have not been widely applied to this area, leaving critical gaps in our understanding of lung preservation temperature during SIS [9]. Given the unique anatomy of the lungs, comprising an extensive alveolar surface area and a rich capillary network, temperature gradients can have a profound impact on tissue viability. Meanwhile, storage in ice creates an environment that poses risk of freezing injury, limiting the safe ischemic time to 6–8 h [10,11,12,13,14].

In this study, we revisit the fundamental premise that “ice is 4 °C” by systematically measuring the actual temperature profiles of donor lungs in standard SIS. We hypothesize that lung temperatures under SIS commonly drop below 4 °C, sometimes nearing the freezing point, thereby increasing the risk of freezing injury. Through a combination of preclinical experiments and clinical observations, we aim to elucidate the true range of organ temperatures encountered in SIS.

## 2. Methods

This is a combined porcine and clinical study.

### 2.1. Porcine

**Figure 1:**
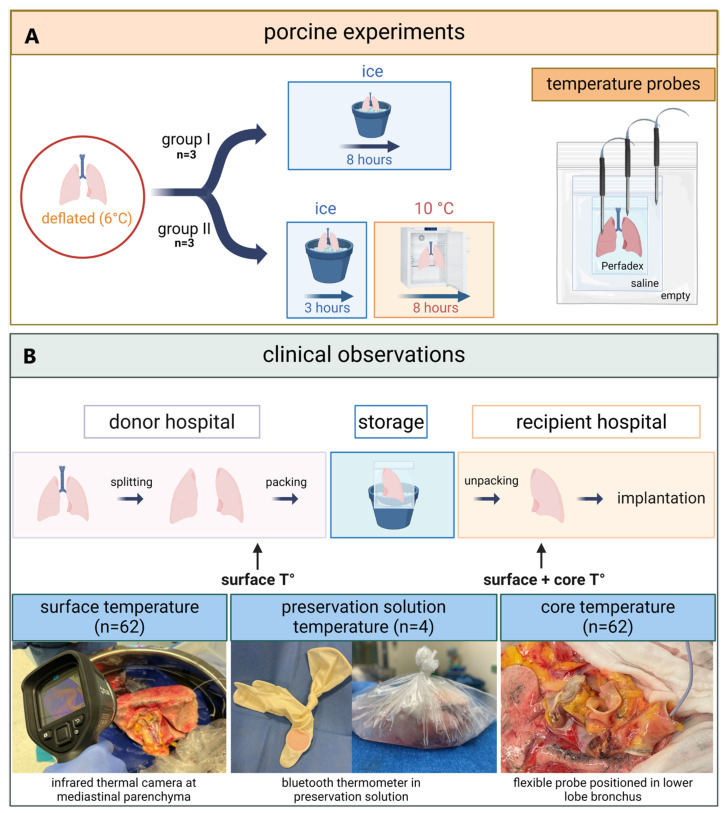
(**A**) Porcine lungs were frozen and delivered to the Paragonix laboratories followed by deflation and rewarming to 6 °C. Subsequently, they were enrolled in two thermodynamic evaluation protocols. The first protocol (group I) consisted of 8 h of storage in ice while the second protocol (group II) stored lungs for 3 h in ice followed by rewarming in a refrigerator (10 °C) for 8 h. Temperature measurements were systematically recorded. (**B**) Donor lungs were procured in the donor hospital followed by separation. Surface temperature (surface T°) was measured using an infrared thermal camera immediately before and after SIS. After SIS, an additional core temperature (core T°) measurement was performed.

The preclinical study on porcine lungs (Figure 1) was carried out by researchers from Paragonix Technologies. Six porcine double-lung blocks, procured by a commercial medical supplier (Sierra for Medical Science), were delivered in a frozen state to the laboratory. Double-lung blocks were used for each experiment. The pig lungs were thawed to a temperature of 6 °C and packed in three bags as recommended by the International Society for Heart and Lung Transplantation (ISHLT) [15]. The lungs were left deflated, resulting in a lower surface-to-mass ratio and reduced thermal conductivity, which may have slowed cooling. The inner bag contained Perfadex (XVIVO, Mölndal, Sweden) preservation solution, the middle bag contained saline 0.9% NaCl with no added slushed ice, and the outer bag remained empty. The lung blocks were allocated to two study arms during which temperature was systematically recorded using hardwired temperature probes (CX402-xxM, InTemp, Bourne, MA, USA). A sharp probe was inserted in the right or left lower lobe at a 2.5 cm depth to measure tissue temperature (tissueT°). A second probe was positioned in the inner bag to measure preservation solution temperature (psT°). The third probe was positioned in the second bag to measure saline temperature (salineT°). Temperature was measured at 30 s intervals throughout the experiment.

Three lung blocks were allocated to the first study group (group I) of 8 h of SIS. After packaging and placement of temperature probes (cf. supra), porcine lungs were placed in an icebox. One saline monitoring experiment was excluded from the analysis due to an incorrect calibration of the temperature probe. The remaining three lung blocks were allocated to the second study group (group II) with 3 h of SIS followed by 8 h of storage in a standard 10 °C laboratory refrigerator.

### 2.2. Clinical

The clinical observational study was performed at University Hospitals Leuven, and an overview is shown in Figure 1. This study was conducted in accordance with the Declaration of Helsinki, and the protocol was approved by the Ethics Committee UZ/KU Leuven (S67052) on 28 November 2022. Written informed consent was obtained from all recipients.

#### 2.2.1. Preservation Solution Temperature During Static Ice Storage in Clinical Lung Transplantation

We longitudinally measured psT° during the SIS of four left donor lungs. Standard practice for SIS, as recommended by the ISHLT, was followed [13]. Lungs were split in the donor center and individually packed in three bags. The first bag contained 1 L of Organ Care System (OCS, Transmedics, Andover, MA, USA) lung preservation solution. The second bag contained 1 L of saline solution with added slushed ice. The third, outer bag remained empty. OCS and saline solutions were stored on ice prior to packing. To measure psT° during SIS, a Tempo Disc Bluetooth thermometer (Blue Maestro, London, UK) was put in two surgical gloves and positioned in the inner bag filled with OCS. At 1 min intervals, psT° was measured during SIS until the lung was removed from the bag for implantation. The recorded psT° data were transferred to the Tempo Plus 2 application (Blue Maestro, London, UK) for analysis.

#### 2.2.2. Surface and Core Temperature of Human Donor Lungs Before and After Static Ice Storage

Surface (surfaceT°) and core (coreT°) temperatures were measured in clinical donor lungs stored using standard SIS protocols (cf. supra). Thirty-one sequential sing-lung transplants were performed, resulting in 62 lungs for which temperatures were measured. First, surfaceT° was measured just prior to packing of the lungs in the donor center. Immediately after unpacking the lung at the end of SIS in the transplant center (within 5 min), surfaceT° and coreT° were measured. Lungs were individually unpacked in the transplant center according to the implantation sequence. SurfaceT° was measured with an E8 FLIR infrared thermal camera (Teledyne, Lincoln, NE, USA) directed at the mediastinal lung parenchyma. coreT° was measured with a 6Fr flexible temperature probe (SKU 22-GP406-S, Alleset, Jiaxing, China), immediately wedged in the lower lobe bronchus after removing the stapler line.

### 2.3. Statistics

For porcine experiments, data are presented as the mean ± standard error of the mean (SEM), derived from the average of results obtained from three independent experiments.

Clinical donor characteristics were summarized using the median ± interquartile range (IQR) for continuous data (age body mass index (BMI), ventilator time, PaO_2_/FiO_2_ ratio, cold ischemic storage duration) and were summarized with observed frequencies and percentages (%) for categorical data (sex, donor type). surfaceT° and coreT° were summarized as the median (IQR) and plotted with Graphpad Prism 10 (Prism, Boston, MA, USA).

## 3. Results

### 3.1. Porcine Lung Temperature Evolution During Static Ice Storage

**Figure 2:**
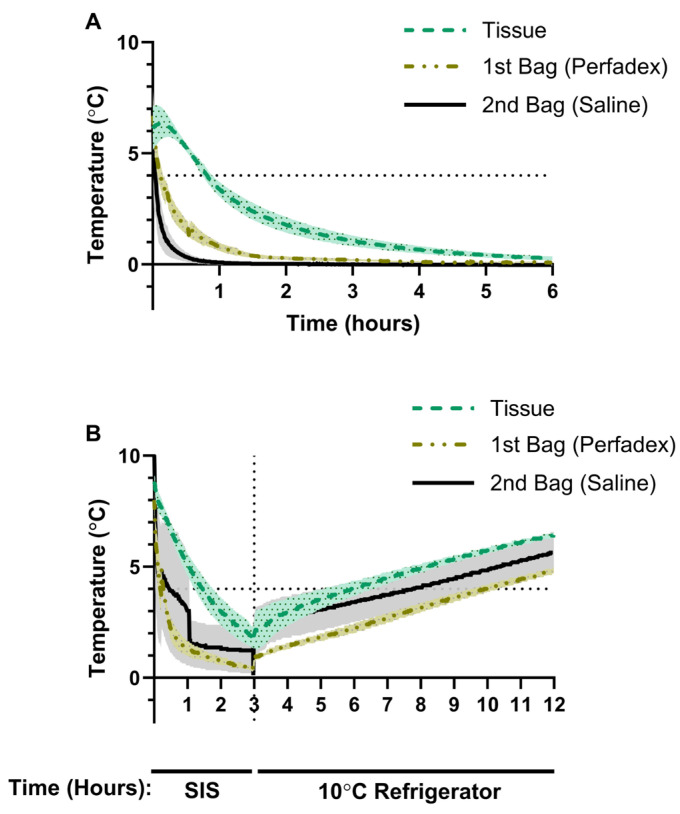
(**A**) Group I changes in the temperature of saline solution (*n* = 2), Perfadex solution (*n* = 3) and lung tissue (*n* = 3) in porcine lungs stored using static ice storage (SIS). (**B**) Group II changes in the temperature of saline solution, Perfadex solution and lung tissue in porcine lungs preserved for 3 h using SIS before transfer to a refrigerator set to 10 °C (*n* = 3). Data are presented as the average temperature ± SEM.

At the start of SIS in group I, average tissueT° was 6.11 °C, psT° was 6.70 °C and salineT° was 7.08 °C (Figure 2A). After 1 h, the mean tissueT° dropped below 4 °C, reaching 3.38 °C, before continuing to decrease to 1.77 °C after 2 h and 0.66 °C after 4 h (Figure 2A). psT° dropped rapidly to 0.80 °C after 1 h, 0.28 °C after 2 h and 0.12 °C after 4 h (Figure 2A). salineT° dropped to a temperature of 0.08 °C after 1 h and dropped below 0 °C after 2.25 h, with the lowest mean temperature of −0.03 °C being recorded over the 8 h observation period (Figure 2A).

### 3.2. Porcine Lung Temperature During Static Ice Storage Followed by Storage at 10 °C

In group II, we investigated how porcine lung temperature changed when exposed to 10 °C following a 3 h period of SIS. After SIS and prior to transfer to the 10 °C refrigerator, average lung tissueT° was 1.90 °C, psT° was 0.57 °C and salineT was 1.57 °C (Figure 2B). Exposure to 10 °C resulted in a gradual temperature increase with a maximal average tissueT° of 6.47 °C, psT° of 4.83 °C and salineT° of 5.63 °C (Figure 2B). Time to reach 4 °C after SIS followed by 10 °C exposure was 2.90 h for tissueT°, 6.90 h for psT° and 7.73 h for salineT° (Figure 2B). Over the 8 h rewarming period, none of the temperature probes recorded a measurement of 10 °C (Figure 2B).

### 3.3. Longitudinal Monitoring of Preservation Solution Temperature in Human Donor Lungs

**Figure 3:**
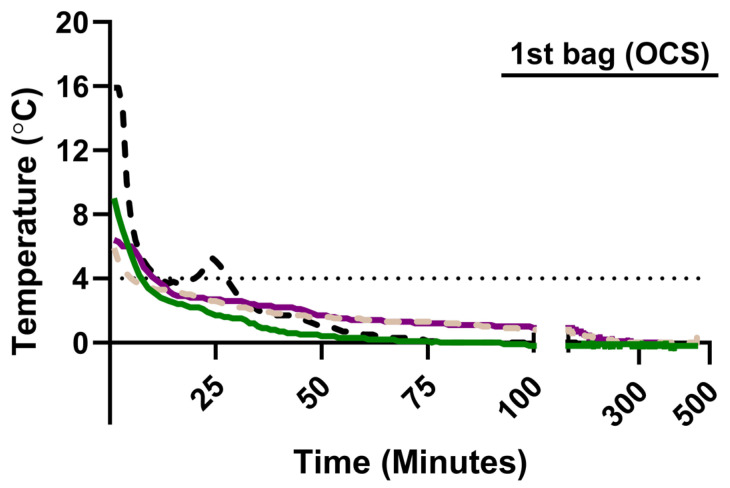
Longitudinal changes in the temperature of the preservation solution (OCS) surrounding the lung during static ice storage of human donor lungs. Each line represents data from an individual donor lung (*n* = 4).

Measurement of psT° with the Bluetooth thermometer in OCS showed that the initial psT° at the start of SIS is heterogenous—ranging from 5.9 °C to 15.9 °C—but also that the psT° dropped below 4 °C within 9–13 min of SIS (Figure 3). In two cases, psT° dropped to 0.0 °C after 78 min and remained at or below 0.0 °C for the duration of SIS (Figure 3). In two other cases, psT° dropped to 1.0 °C within 91 min before gradually decreasing to 0.0 °C after 259 and 267 min.

### 3.4. Human Donor Lung Temperature Before and After Static Ice Storage

To study the effect of SIS on organ temperature in clinical LTx cases, we measured surfaceT° and coreT° before and after SIS temperatures in donor lungs. The donor characteristics are shown in Table 1.

**Figure 4:**
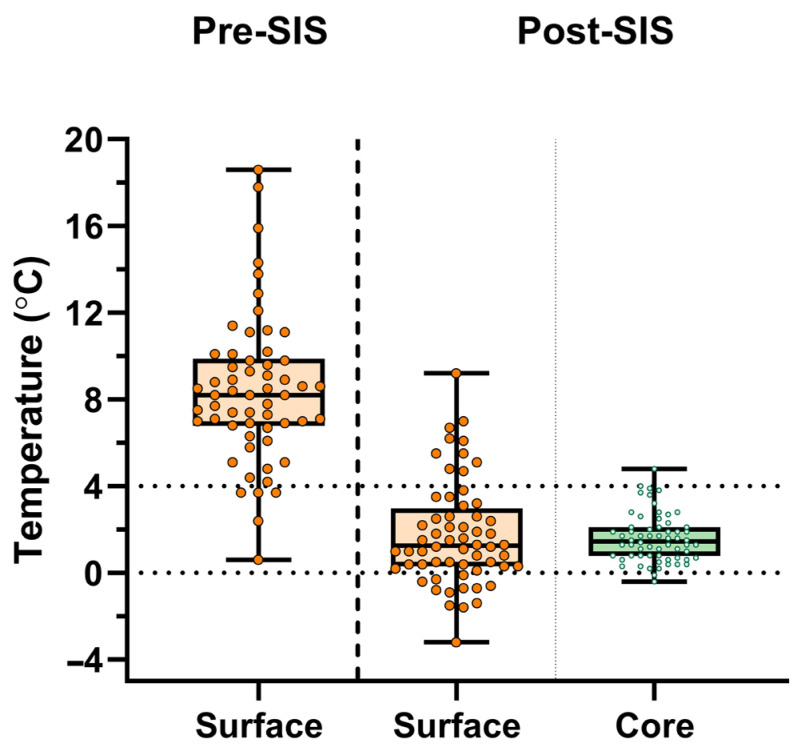
Surface temperature of 62 donor lungs just prior to packing followed by static ice storage (pre-SIS). Surface (orange) and core (green) temperature after unpacking at the end of SIS (post-SIS surface; and post-SIS core). Data are presented as the median ± interquartile range, with each point representing measurement data of individual human donor lungs.

Donor lungs from 31 double-LTx procedures (*n* = 62 lungs) were measured at each timepoint. The average donor age was 59.0 ± 20.0 years, 54.8% were male, and 48.4% were donations after circulatory death (DCD-III). Prior to lung packing, median surfaceT° was 8.2 °C (6.78–9.88 °C). Median SIS duration was 254 min (min-max; 79–609). Immediately after unpacking in the transplant center, median surfaceT° was 1.25 °C (0.30–2.98 °C) and median coreT° was 1.45 °C (0.78–2.10 °C) (Figure 4).

## 4. Discussion

We successfully measured SIS lung temperatures in both preclinical and clinical contexts using an accurate, easy to use and highly versatile approach that can serve as a valuable tool for future clinical studies. Our findings challenge the long-standing assumption that SIS maintains lungs at 4 °C, demonstrating that temperatures can fall below 4 °C and reach 0 °C, thereby risking freezing injury. Although substances generally consolidate as they reach their maximum density, water is an exception to this rule. While 4 °C represents the temperature at which water achieves its maximum density, its actual freezing point at atmospheric pressure is 0 °C [16] (Supplementary Figure S1). Saline (0.9% NaCl), commonly used in the second preservation bag, has a freezing point of approximately −0.59 °C, while Robicsek et al. observed that saline ice temperatures are as low as −7.1 °C in the clinical cardiac surgery setting, with slush ice forming at −0.6 °C [17].

Organ temperature during preservation depends on several factors, including the quantity of preservation bags, the presence of ice slush in the second bag, the volume of preservation solution and the preservation duration. In the preclinical study, we show that cooling occurs centripetally, where the temperature of the outer layer bags cool down first. Nevertheless, SIS, particularly in the clinical setting, poses a great risk of freezing injury, especially in donor organs with extended durations of preservation. In our clinical setting, psT° dropped to 0 °C rapidly in one case after 97 min, while in other cases it decreased more gradually, eventually reaching 0 °C. Furthermore, post-SIS temperatures, particularly for surfaceT°, had a large variability, with temperatures below 0 °C. Previously, Horch et al. showed that organs stored on ice averaged 2 °C, reaching 0 °C by 6 h [18]. Several studies demonstrated that cold-induced injury associated with SIS can directly reduce vascular muscle function [6,7], promote vascular edema [19] and compromise mitochondrial viability and ATP levels, causing increased reactive oxygen species (ROS) levels [20]. Direct freezing injury can potentially denature proteins and disrupt protoplasmic structures at the cell surface [17]. Formation of ice crystals at near-freezing temperatures can result in electrolyte and osmotic imbalances, leading to organelle swelling and mitochondrial damage [21,22]. Taken together, avoiding freezing injury by limiting SIS to 6 h has been advised [10,11,12,13,14].

Recently, the field of lung preservation has seen revolutionary advances with the introduction of controlled hypothermic storage (CHS) devices [23]. These systems maintain lungs at 4–10 °C without the use of ice, thereby avoiding freezing injury. Moreover, CHS allows for a safe extension of lung CIT, introducing flexibility for transplant clinicians without compromising outcome [24,25]. It reduces time constraints and logistical challenges, primarily encountered in the context of distant procurements and complex cases. A 10 °C lung preservation has earlier been suggested as an alternative to SIS and to result in better physiological function, mitochondrial health and antioxidative profiles while avoiding the risk of freezing injury. A clinical proof of concept in five hybrid SIS/CHS cases demonstrated overnight bridging of LTx to daytime hours, and a larger multicenter trial comparing 70 CHS to 140 propensity-matched SIS cases reported no significant differences in PGD3 at 72 h, 30-day survival, or 1-year survival [20,26]. However, the lack of a transportable device capable of maintaining strict 10 °C preservation led to the use of hybrid preservation strategies in the previous studies, using SIS transport followed by storage in a 10 °C incubator [20,26]. In our study, after three hours of SIS, subsequent storage at 10 °C for eight hours led to only a gradual increase in temperature, falling short of reaching 10 °C. The previously reported clinical data may therefore not represent the full potential of CHS for improved lung preservation and implies potential freezing injury during transportation. The impact of this initial freezing step, which was not included in the initial preclinical experiments, warrants further investigation [14,27,28,29].

To ensure optimal preservation temperature from procurement to implantation, CHS devices should therefore be transportable. Several devices have been developed: LUNGguard, Xport and Vitalpack [23]. We previously showed that in a multicenter observational study, extending the lung preservation of 13 double-lung transplants beyond 15 h appears to be safe for applying CHS with LUNGguard. This is a transportable device, maintaining temperature at 4–8 °C [24]. The average preservation temperature in this study was 7 °C. Although this was a high-risk recipient cohort, including three high-urgent patients, no PGD3 at 72 h was observed.

In the new era of CHS, it is important to appreciate the distinction between the actual organ and preservation device temperature and their effect on clinical outcome. Our findings advocate the importance of the correct measurement and reporting of organ temperatures in future experimental and clinical research.

Our study is limited by potential variations in temperature probes that could arise due to numerous factors, including minor differences in calibration. Regarding the porcine study, porcine lungs were procured, frozen-delivered and then thawed and deflated. The deflated state of these lungs results in reduced surface area in contact with the preservation solution, which lowers thermal conductivity. Despite this, the total lung mass difference in deflated vs. inflated lungs are negligible in terms of thermal impact (e.g., inflated 6 L of air corresponding to a mass of 7.76 g). Thus, this preclinical study represented an overestimation of temperatures reached with SIS. However, the results were comparable with those collected during the clinical portion of this study. The clinical study is limited by the absence of a control group, ideally one using CHS, necessitating future comparisons of temperature variability and clinical outcomes. For longitudinal analyses in human LTx, we only recorded longitudinal measurements for psT° and not for the temperature of the donor lungs. An uncontrollable variable in the clinical study was preservation duration, which might have contributed to a large variation in post-SIS surfaceT° and coreT°. Additionally, the placement of an endobronchial probe provides an approximate measurement of the coreT°, but it is not feasible to directly measure the temperature of the tissue without causing iatrogenic injury by inserting a probe in the parenchyma. It is noteworthy to address that not all centers use the same packing methods. While some centers pack double lungs together, our center prefers to pack right and left lungs separately at the procurement center.

## 5. Conclusions

This study provides the first clinical evidence that SIS does not reliably maintain donor lung temperature at 4 °C, instead exhibiting marked variability and unpredictable post-preservation temperatures. These findings highlight the need to carefully weigh the benefits against the risks of SIS, particularly in extended-criteria donors where prolonged ischemic times at suboptimal temperatures can affect organ quality. Future studies should evaluate whether temperature variability is associated with graft survival, post-transplant complications or inflammatory responses, with the goal of improving lung preservation protocols.

## Figures and Tables

**Table 1:** Lung transplant donor characteristics. Data are presented as the median ± interquartile range or percentage where indicated. PaO_2_: Partial pressure of arterial oxygen; FiO_2_: fraction of inspired oxygen.

Transplant cases using SIS	31
Number of lungs stored in ice	62
Age (years)	59.0 (48–68)
Sex (%male)	54.8
Donor type (DCD III)	48.4
Body mass index (kg/m^2^)	25.0 (23–29)
Ventilator time (hours)	146.0 (75–246)
Donor P/F ratio (PaO_2_/FiO_2 100%_)	431 (386–507)
Cold ischemic time (hours)	4 h 12 (2 h 43–5 h 19)

## Data Availability

The data presented in this study are available on request from the corresponding author due to the specific format of data presentation (continuous temperature measurement).

## Supplementary Materials

The following supporting information can be downloaded at: https://www.mdpi.com/article/10.3390/jcm14062127/s1, Figure S1: Phase transition behavior of water.

## Abbreviations

The following abbreviations are used in this manuscript:

CHS	Controlled hypothermic storage
coreT°	Core temperature
FiO_2_	Fraction of inspired oxygen
ISHLT	International Society for Heart and Lung Transplantation
LTx	Lung transplantation
OCS	Organ Care System preservation solution
psT°	Preservation solution temperature
PaO_2_	Partial pressure of arterial oxygen
PGD3	Primary graft dysfunction grade 3
ROS	Reactive oxygen species
salineT°	Saline temperature
SIS	Static ice storage
surfaceT°	Surface temperature
tissueT°	Tissue temperature
