# Supplementary material for: Temperature Dynamics of Porcine and Human Lungs During Static Ice Storage: Ice Is Not 4 °C
Source: J Clin Med. 2025 Mar 20;14(6):2127. doi: 10.3390/jcm14062127 (PMC11943378; doi:10.3390/jcm14062127)
Supplement: Supplementary file 1 [file jcm-14-02127-s001.zip › jcm-3443628-supplementary.pdf]

Supplementary Materials

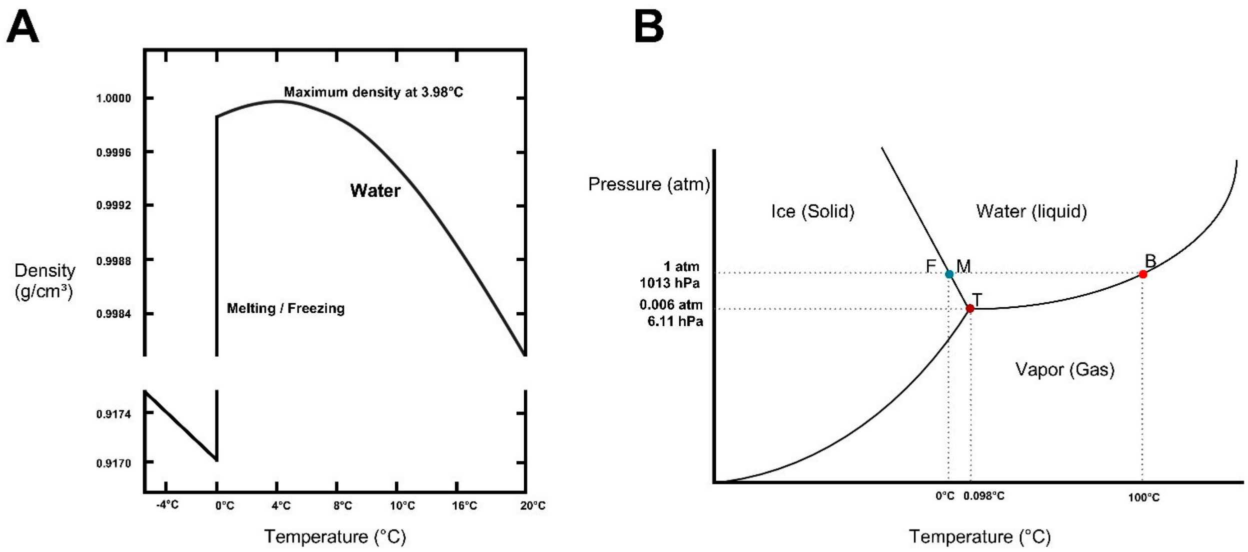

Figure S1. Physical properties of water. (**A**) Density of water/ice as a function of temperature. Note the maximum density of water at 4 °C and the freezing (F) or melting (M) point of water at 0 °C. (**B**) Phase diagram of water illustrating the triple point (T), boiling point (B) and freezing (F) or melting (M) point as a function of pressure (P) and temperature. For 1013 hPa, corresponding to the atmospheric pressure (atm), the freezing point of water is at 0 °C and boiling point of water is at 100 °C.
